# Personality profiles and coaching leadership preferences among Chinese female basketball players: a latent profile analysis

**DOI:** 10.3389/fpsyg.2026.1909620

**Published:** 2026-09-10

**Authors:** Jiahui Ye, Xiang Pan

**Affiliations:** 1 School of Physical Education and Training, Capital University of Physical Education and Sport, Beijing, China; 2 China Basketball Academy, Beijing Sport University, Beijing, China

**Keywords:** Big Five personality traits, coach–athlete relationship, coaching leadership behavior, female basketball players, latent profile analysis

## Abstract

**Introduction:**

Athletes' personality is an important factor influencing their preferences for coaches' leadership behaviors. However, previous studies have primarily adopted a variable-centered approach and have rarely examined these preferences from a person-centered perspective. This study aimed to identify latent personality profiles among Chinese female basketball players and examine how these profiles, together with individual personality traits, were associated with preferences for different coaching leadership behaviors, thereby providing practical implications for coaching practice.

**Methods:**

Female athletes from the Women's Chinese Basketball Association (WCBA) and the Chinese University Basketball Association (CUBAL) completed measures of the Big Five personality traits and preferences for coaching leadership behaviors. Latent profile analysis (LPA) was conducted to identify personality profiles, followed by analyses of profile differences in leadership behavior preferences and regression analyses to examine the associations between personality traits and coaching leadership preferences within each profile.

**Results:**

Three personality profiles were identified: Sensitive, Average, and Resilient. Across all three profiles, athletes showed the strongest preference for training and instruction, followed by positive feedback, democratic behavior, social support, and autocratic behavior. Except for autocratic behavior (*F* = 0.02, *p* = 0.98, ηp^2^ = 0.00), preferences for all other leadership behaviors differed significantly across personality profiles (all *p* < 0.01). Within the Sensitive profile, conscientiousness was negatively associated with preferences for training and instruction, autocratic behavior, and social support, whereas agreeableness was positively associated with preferences for training and instruction, social support, and positive feedback. Neuroticism was positively associated with preferences for positive feedback. Within the Average profile, extraversion was positively associated with preferences for democratic behavior and positive feedback, whereas neuroticism was positively associated with preferences for autocratic behavior. Within the Resilient profile, conscientiousness was positively associated with preferences for social support.

**Discussion:**

The associations between personality traits and preferences for coaching leadership behaviors differed across personality profiles, indicating that athletes with similar trait levels may exhibit distinct leadership preferences depending on their overall personality configuration. These findings highlight the value of a person-centered approach for understanding athletes' coaching leadership preferences and provide practical implications for developing more individualized coaching strategies.

## 1 Research background

The multidimensional model of coaching leadership extends general leadership theories to the sport context by emphasizing that leadership effectiveness arises from the dynamic interaction among coach characteristics, athlete characteristics, and situational factors (Chelladurai, 1980). Drawing on contingency theory, path-goal theory, and life-cycle theory, this framework proposes that coaching behaviors should be evaluated not only in terms of their direct effects on performance outcomes but also in relation to their alignment with athletes' psychological needs and expectations. Empirical research has shown that coaching leadership behaviors are associated with athlete satisfaction, motivation, commitment, and team cohesion (Kenow and Williams, 1999; Wrisberg, 1996; Wałach-Biśta, 2019).

Within this framework, the congruence between athletes' preferred coaching behaviors and coaches' actual leadership behaviors is considered a key determinant of effective coach–athlete interactions. When coaches' leadership behaviors align with athletes' expectations, athletes are more likely to experience greater psychological engagement and develop stronger relationships with their coaches (Kenow and Williams, 1999; Wrisberg, 1996). Accordingly, identifying the individual factors that influence athletes' leadership preferences is important for developing more adaptive and personalized coaching strategies. Among these factors, personality is a core psychological characteristic that influences how athletes perceive, interpret, and respond to interpersonal interactions. Defined as a relatively stable pattern of cognitive, emotional, and behavioral tendencies (Chen and Zhang, 1988), personality provides an important theoretical foundation for understanding individual differences in preferences for coaching leadership behaviors.

Despite growing scholarly interest in coaching leadership, the psychological mechanisms through which athlete characteristics influence preferences for coaches' leadership behaviors remain insufficiently understood. Previous research has primarily focused on demographic and contextual factors, such as cultural background and gender, as determinants of leadership preferences. However, findings on gender differences have been inconsistent (Sherman et al., 2000), suggesting that demographic characteristics alone may have limited explanatory value. One possible explanation is that such approaches overlook the psychological heterogeneity among athletes who share similar demographic characteristics.

This issue may be particularly relevant among female athletes in team sports. Previous research suggests that coaches often perceive interactions with female athletes as involving greater emotional and interpersonal demands (Seller and Valley, 1999), while qualitative evidence indicates that female athletes differ considerably in emotional sensitivity and interpersonal expectations (Fasting and Pfister, 2000). In sports such as basketball, where coaches must simultaneously address the diverse psychological and performance needs of multiple athletes, understanding these individual differences is essential for developing effective individualized leadership strategies within a team setting. Nevertheless, research on women's basketball has rarely examined coach–athlete interactions from the athletes‘ perspective, and the role of personality profiles in shaping preferences for coaches' leadership behaviors remains largely unexplored.

The Big Five personality framework provides a well-established framework for examining individual differences in sport psychology. However, previous research has predominantly adopted a variable-centered approach, focusing on the independent effects of individual personality traits on specific outcomes. Although this approach has generated valuable insights, it assumes that personality traits exert relatively consistent effects across individuals and may therefore overlook the way personality traits combine within individuals. In reality, personality traits rarely operate in isolation. Instead, their behavioral effects may depend on the interaction of multiple traits within the same individual. Consequently, an important theoretical question remains: do personality traits influence preferences for coaches' leadership behaviors through universal trait-specific effects, or do these preferences differ across personality profiles that reflect distinct patterns of psychological adaptation?

Latent profile analysis (LPA), a person-centered analytical approach, offers a useful method for addressing this theoretical limitation by identifying naturally occurring personality profiles and examining how these profiles are associated with distinct patterns of preferences for coaches leadership behaviors (Guo and Mao, 2023). Rather than merely classifying athletes into different groups, LPA makes it possible to examine whether different combinations of personality traits reflect distinct psychological profiles underlying athletes' preferences for coaches‘ leadership behaviors. Accordingly, the present study applies LPA to identify personality profiles among Chinese female basketball players and examines whether these profiles are associated with different preferences for coaches' leadership behaviors.

## 2 Method

### 2.1 Participants

This study employed a cross-sectional questionnaire design. Participants were recruited through convenience sampling from two competitive settings in China: the Women's Chinese Basketball Association (WCBA) and teams competing in the Women's National Finals of the Chinese University Basketball Association (CUBAL).

In the Chinese competitive sports system, athletes are officially classified into three performance grades: Second Class, First Class, and Champion Class. These grades are awarded by the General Administration of Sport of China based on standardized criteria, including competition level, athletic performance, and achievements in officially recognized competitions. In women's basketball, higher grades generally reflect a higher level of competitive performance.

A total of 450 questionnaires were distributed, of which 402 were returned. After excluding incomplete questionnaires and those completed in an unreasonably short time, 326 valid questionnaires were included in the final analysis, yielding an effective response rate of 72.4%. Participant characteristics are presented in Table 1.

**Table 1:** Demographic profile of the study participants (*n* = 326).

Characteristic	Category	*n* (%)
Age (years)	18–21	227 (69.6)
	22–24	78 (24.0)
	≥25	21 (6.4)
Athlete classification	Champion class	60 (18.4)
	First class	162 (49.7)
	Second class	104 (31.9)
Competition category	WCBA	105 (32.2)
	CUBAL	221 (67.8)

Participants were eligible if they (a) were female basketball players aged 18 years or older, (b) were active roster members of WCBA or CUBAL teams during the data collection period, and (c) had trained and competed under their current head coach for at least one year.

Participants were recruited through team contacts and coaching staff. Coaches assisted only by facilitating access to their teams and informing athletes about the study. Participation was entirely voluntary, and each athlete decided independently whether to participate. Questionnaires were completed anonymously without coaches being present. Coaches had no access to individual questionnaires and were not informed which athletes participated, thereby protecting participants' confidentiality and minimizing potential response bias.

Before data collection, all participants provided written informed consent. Ethical approval was obtained from the university's Academic Ethics Committee, and all study procedures complied with the ethical standards for research involving human participants.

### 2.2 Instrumentation

#### 2.2.1 Big Five personality

Personality traits were assessed using the Chinese version of the Big Five Inventory−2 (BFI-2), which was revised and validated by the Department of Psychology at Texas A&M University and the Faculty of Psychology at Beijing Normal University (Zhang et al., 2021). The inventory consists of 60 items assessing five personality dimensions: extraversion, agreeableness, conscientiousness, neuroticism, and openness to experience. Each dimension comprises three facets, with four items per facet and a balanced number of positively and negatively worded items. Previous validation studies supported the cross-cultural validity of the Chinese BFI-2 through exploratory structural equation modeling and confirmatory factor analysis (Zhang et al., 2021). In the present study, Cronbach's α coefficients were 0.81 for Extraversion, 0.82 for Agreeableness, 0.88 for Conscientiousness, 0.82 for Neuroticism, and 0.78 for Openness to Experience. The overall scale demonstrated good internal consistency (Cronbach's α = 0.86).

#### 2.2.2 Leadership scale for sports

Coaching leadership preferences were assessed using the athlete preference version of the Leadership Scale for Sports (LSS), originally developed by Chelladurai and Saleh and later revised and validated for Chinese athletes (Gao et al., 2021). The validated Chinese version of the LSS was used in the present study.

The Chinese LSS consists of 15 items measuring five dimensions of coaching leadership behavior: training and instruction, democratic behavior, autocratic behavior, social support, and positive feedback, with three items for each dimension. All items begin with the stem “I prefer my coach to …” (e.g., “provide clear technical instruction during training”) and are rated on a 5-point Likert scale ranging from 1 (never) to 5 (always).

Previous studies have demonstrated satisfactory construct validity and internal consistency of the Chinese LSS, with confirmatory factor analysis supporting the original five-factor structure (Gao et al., 2021). In the present study, Cronbach's α coefficients were 0.84 for Training and Instruction, 0.77 for Democratic Behavior, 0.76 for Autocratic Behavior, 0.83 for Social Support, and 0.90 for Positive Feedback, indicating acceptable to good internal consistency across all dimensions.

### 2.3 Statistical analysis

#### 2.3.1 Latent profile analysis

Given the multidimensional nature of personality and the interrelationships among the Big Five personality traits, latent profile analysis (LPA), a person-centered approach, was conducted to identify naturally occurring personality profiles among Chinese female basketball players. Compared with traditional variable-centered approaches, LPA identifies homogeneous latent subgroups based on individuals' response patterns across multiple personality traits, providing a more comprehensive understanding of personality heterogeneity.

LPA was conducted using Mplus 8.0 based on the five personality traits (Extraversion, Agreeableness, Conscientiousness, Neuroticism, and Openness to Experience). Models specifying one to five latent profiles were estimated. Model fit was evaluated using the Akaike information criterion (AIC), Bayesian information criterion (BIC), sample-size adjusted Bayesian Information Criterion (aBIC), entropy, and the Lo-Mendell-Rubin likelihood ratio test (LMR-LRT) (Xie et al., 2019). Lower AIC, BIC, and aBIC values, higher entropy values, and a significant LMR-LRT (*p* < 0.05) indicated better model fit. Following previous recommendations, the final model was selected based on overall model fit, entropy values greater than 0.80, profile interpretability, and adequate profile size. The identified personality profiles were subsequently used to examine differences in preferences for coaches' leadership behaviors and profile-specific associations between personality traits and these preferences.

#### 2.3.2 BCH comparisons and profile-specific regression analyses

To compare preferences for coaches‘ leadership behaviors across the identified personality profiles while accounting for classification uncertainty, the Bolck-Croon-Hagenaars (BCH) three-step procedure was implemented in Mplus 8.0. The five dimensions of coaches' leadership behaviors were specified as continuous distal outcomes. The BCH procedure estimates profile-specific means while adjusting for classification error and is recommended for examining differences in distal outcomes following latent profile analysis.

Following the BCH analyses, profile-specific multiple linear regression analyses were conducted to examine whether the associations between the Big Five personality traits and preferences for coaches‘ leadership behaviors differed across the identified personality profiles. Separate regression models were estimated for each personality profile, with the five personality traits entered simultaneously as predictors and the five dimensions of coaches' leadership behaviors treated as dependent variables.

Prior to model estimation, multicollinearity was assessed. Variance inflation factor (VIF) values below 2.0 and tolerance values above 0.50 indicated no evidence of problematic multicollinearity.

#### 2.3.3 Assessment of common method variance

Because all study variables were measured using self-report questionnaires administered at a single time point, common method variance (CMV) was considered a potential source of bias.

As a preliminary assessment, all items from the Big Five Personality Inventory and the Coaching Leadership Behavior Preference Scale were entered into an unrotated exploratory factor analysis, and Harman's single-factor test was performed. The analysis yielded 20 factors with eigenvalues greater than one, accounting for 66.58% of the total variance. The first unrotated factor explained 18.45% of the total variance, well below the commonly used threshold of 40% (Podsakoff et al., 2003). These results suggest that CMV was unlikely to pose a substantial threat to the study findings.

## 3 Result

### 3.1 Personality profile classification and characteristics

Latent profile analysis (LPA) was conducted using the standardized scores of the five Big Five personality traits (extraversion, agreeableness, conscientiousness, neuroticism, and openness to experience) from 326 female basketball players. Model fit indices for the competing profile solutions are presented in Table 2.

**Table 2:** Information of potential profile fitting indicators of “Big Five” personality.

Category	Fitting indicators					
	AIC	BIC	aBIC	Entropy	LMRT	BLRT
Class 1	2452.839	2490.708	2458.988	–	–	–
Class 2	2058.418	2119.009	2068.258	0.819	0.000	0.000
Class 3	1941.512	2024.821	1955.042	0.801	0.006	0.000
Class 4	1904.988	2011.021	1922.212	0.793	0.037	0.000
Class 5	1886.822	2015.576	1907.734	0.828	0.076	0.000

Solutions specifying one to five latent profiles were estimated, and the corresponding model fit indices are presented in Table 2. Except for the one-profile solution, all models yielded significant BLRT results (*p* < 0.001), indicating that models with multiple profiles provided a better fit than the single-profile solution. Although the AIC, BIC, and adjusted BIC values continued to decrease as the number of profiles increased, the magnitude of these improvements gradually diminished. Moreover, the five-profile solution was not supported by the LMR-LRT (*p* = 0.076), suggesting that adding a fifth profile did not significantly improve model fit relative to the four-profile solution.

Although the four-profile solution showed slightly lower information criteria and a significant LMR-LRT, model selection was not based solely on statistical fit indices. Consistent with recommendations for latent profile analysis (Nylund et al., 2007), profile selection also considered classification quality, profile size, theoretical interpretability, and practical utility. Compared with the three-profile solution, the four-profile solution produced a very small profile, which may reduce the stability of parameter estimates and limit the robustness of subsequent regression analyses. In addition, the additional profile primarily represented a subdivision of an existing profile rather than a substantively distinct personality pattern, reducing model parsimony without providing meaningful theoretical advantages.

The three-profile solution demonstrated acceptable classification accuracy (entropy = 0.801), significant LMR-LRT and BLRT results, adequate profile sizes for subsequent analyses, and conceptually distinct personality profiles. Taken together, the statistical evidence, theoretical interpretability, model parsimony, and practical considerations supported the selection of the three-profile solution for all subsequent analyses.

To further evaluate the classification accuracy of the latent profile analysis (LPA) solution, the average posterior probabilities (AvePPs) for each latent profile were calculated, and the results are presented in Table 3. The three-profile model demonstrated good classification accuracy. Specifically, individuals assigned to profile C1 showed an average posterior probability of 0.830 for membership in C1, with a probability of 0.170 of being assigned to C2. Individuals classified into C2 exhibited an average posterior probability of 0.898, with relatively low probabilities of assignment to C1 (0.032) and C3 (0.070). Similarly, individuals assigned to C3 showed a high average posterior probability of 0.868 for membership in C3, indicating a low likelihood of alternative profile assignment.

**Table 3:** Average posterior probabilities of latent profile membership.

Profile membership	Category		
	C1	C2	C3
C1	0.830	0.170	0.000
C2	0.032	0.898	0.070
C3	0.000	0.132	0.868

Overall, the average posterior probabilities for all three profiles exceeded 0.80, satisfying the commonly recommended criterion for evaluating classification quality in LPA. These results suggest that the three-profile model provides adequate differentiation among latent groups and demonstrates satisfactory classification stability. Therefore, the identified profiles were considered appropriate for subsequent analyses.

#### 3.1.1 Personality profile category characteristics

To further examine differences in Big Five personality traits across the three personality profiles, one-way ANOVAs were conducted with personality profile membership as the independent variable and the scores of the Big Five traits as dependent variables. In addition, repeated-measures ANOVA was performed, with personality profile membership as the between-subjects factor and personality traits as the within-subjects factor. The results are presented in Table 4.

**Table 4:** Distribution characteristics of the “Big Five” personality profile (*n* = 326).

Variable	Latent categories of personality traits			*F*	* $\eta_{p}^{2}$ *
	profile 1 (*n* = 160)	profile 2 (*n* = 135)	profile 3 (*n* = 31)		
Extraversion	2.95 ± 0.40	3.33 ± 0.41	3.99 ± 0.40	94.90^***^	0.37
Agreeableness	3.56 ± 0.32	4.10 ± 0.30	4.55 ± 0.31	192.25^***^	0.54
Conscientiousness	3.21 ± 0.36	3.89 ± 0.37	4.57 ± 0.31	246.19^***^	0.60
Neuroticism	3.20 ± 0.42	2.66 ± 0.36	1.89 ± 0.39	173.28^***^	0.52
Openness	3.16 ± 0.40	3.44 ± 0.36	4.08 ± 0.39	81.15^***^	0.33

^***^p < 0.001.

Results indicated significant differences across all Big Five personality traits among the three profiles. *Post-hoc* comparisons showed that participants assigned to Profile 3 had the highest levels of Extraversion (3.99 ± 0.40), Agreeableness (4.55 ± 0.31), Conscientiousness (4.57 ± 0.31), and Openness to Experience (4.08 ± 0.39), followed by Profile 2, whereas Profile 1 exhibited the lowest scores.

**Figure 1:**
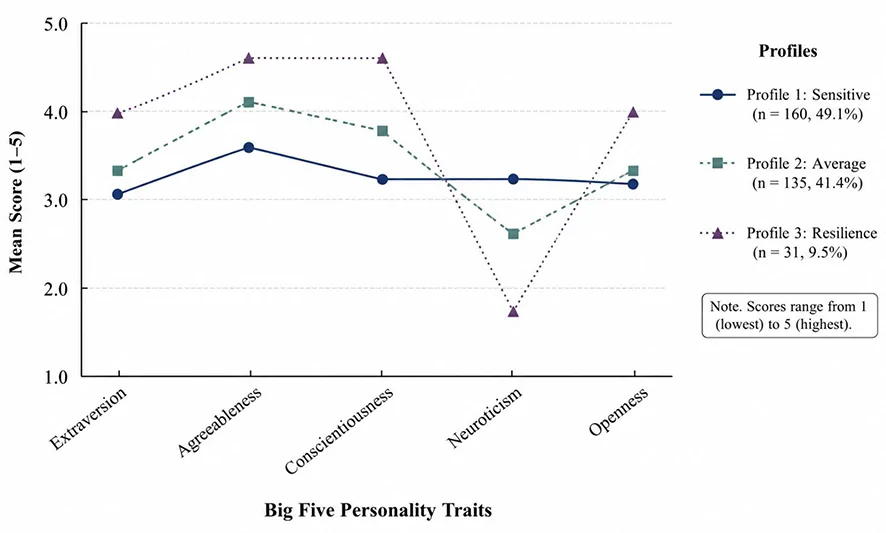
Average scores of different categories in the distribution of “Big Five” personality traits.

As illustrated in Figure 1, the mean scores of the Big Five personality traits demonstrated distinct patterns across the three profiles. Specifically, Extraversion, Agreeableness, and Conscientiousness followed a descending order of Profile 3 > Profile 2 > Profile 1, whereas Neuroticism showed the opposite trend (Profile 1 > Profile 2 > Profile 3). Profile 3 was characterized by the highest levels of positive personality traits and the lowest Neuroticism, representing a more resilient personality pattern. Profile 2 exhibited intermediate levels across most dimensions, whereas Profile 1 showed comparatively elevated Neuroticism and relatively lower scores on other traits, indicating a more balanced but less pronounced personality profile.

#### 3.1.2 Personality profile category naming

Based on the results of the latent profile analysis, the three profiles were labeled and interpreted according to their characteristic patterns of Big Five personality traits. The names and defining characteristics of the three personality profiles are summarized in Table 5.

**Table 5:** Names of different personality profiles and summary of individual characteristics.

profile	Characteristics	Profile naming	Author (Year)
1	Higher levels of neuroticism, while other personality traits exhibit relatively lower levels.	Sensitive	Udayar et al. (2020)
2	Moderate levels of various personality traits	Average	Udayar et al. (2020)
3	Lower levels of neuroticism, relatively higher levels of other personality traits	Resilience	Donnellan and Robins (2010)

Individuals assigned to Profile 1 were characterized by significantly higher Neuroticism scores compared with those in the other profiles, resembling the “high-neuroticism/low-adaptability” profile identified by Udayar et al. (2020). Considering the achievement-oriented context of Chinese athletes, Profile 1 was labeled the “Sensitive” profile.Individuals assigned to Profile 2 showed moderate levels across all five personality traits, without particularly prominent strengths or weaknesses. Consistent with the “mean-type” profile described by Udayar et al. (2020), Profile 2 was labeled the “Average” profile. Individuals assigned to Profile 3 exhibited the lowest Neuroticism scores and relatively higher levels across the remaining personality traits. Based on the theoretical characteristics of the “highly adaptive” profile proposed by Donnellan and Robins (2010), Profile 3 was labeled the “Resilience” profile.

To further examine the characteristics of female basketball players across the three profiles, demographic information for each profile is presented in Table 6.

**Table 6:** Basic information of female basketball players in each profile category (*n* = 326).

Variable	Category	Profile 1 (*n* = 160)	Profile 2 (*n* = 135)	Profile 3 (*n* = 31)
		“Sensitive” profile	“Average” profile	“Resilience” profile
Age	18–21	113 (70.6%)	87 (64.4%)	27 (87.1%)
	22–24	42 (26.3%)	35 (25.9%)	1 (3.2%)
	≥25	5 (3.1%)	13 (9.7%)	3 (9.7%)
Athlete classification	Champion class	35 (21.9%)	21 (15.6%)	4 (12.9%)
	First class	82 (51.2%)	69 (51.1%)	11 (35.5%)
	Second class	43 (26.9%)	45 (33.3%)	16 (51.6%)
Competition category	CUBAL	96 (60.0%)	99 (73.3%)	26 (83.9%)
	WCBA	64 (40.0%)	36 (26.7%)	5 (16.1%)

### 3.2 Preferences for coaches' leadership behaviors among female basketball players with different personality profiles

The BCH comparison results are presented in Table 7. The BCH approach was conducted to examine differences in coaches' leadership behavior preferences across personality profiles.

**Table 7:** Differences in coaches' leadership behavior preferences across personality profiles (*n* = 326).

Variable	“Sensitive” profile (*n* = 160)	“Average” profile (*n* = 135)	“Resilience” profile (*n* = 31)	Waldχ^2^	*P*	*Post-hoc* comparison
	M(SE)	M(SE)	M(SE)			
training instruction	4.017 (0.047)	4.640 (0.098)	4.640 (0.108)	28.820	0.000	1 <2 <3
Democratic	3.620 (0.059)	3.842 (0.071)	4.178 (0.156)	13.903	0.001	1 <2;1 <3
Autocratic	2.750 (0.069)	2.729 (0.065)	2.733 (0.155)	0.043	0.979	–
Social support	3.586 (0.066)	3.757 (0.075)	4.080 (0.162)	9.037	0.011	1 <3
Positive feedback	3.868 (0.060)	4.024 (0.063)	4.478 (0.133)	18.097	0.000	1 <3,2 <3

To examine differences in coaches' leadership behavior preferences across personality profiles, the BCH method was applied to account for classification uncertainty in latent profile membership. Significant differences were identified among the three profiles for most leadership behaviors.

For training and instruction behavior, significant differences were observed across profiles (Wald χ^2^ = 28.820, *p* < 0.001). The Resilience profile showed the highest preference scores, followed by the Average and Sensitive profiles (Profile 3 > Profile 2 > Profile 1). Significant differences were also found for democratic behavior (Wald χ^2^ = 13.903, *p* = 0.001), with both the Resilience and Average profiles showing higher preference scores than the Sensitive profile (Profile 3 > Profile 1; Profile 2 > Profile 1). No significant differences were observed for autocratic behavior (Wald χ^2^ = 0.043, *p* = 0.979).

For social support behavior, significant differences were found among profiles (Wald χ^2^ = 9.037, *p* = 0.011), with the Resilience profile showing higher preference scores than the Sensitive profile (Profile 3>Profile 1). Positive feedback behavior also differed significantly across profiles (Wald χ^2^ = 18.097, *p* < 0.001), with the Resilience profile showing higher preference scores than both the Average and Sensitive profiles (Profile 3 > Profile 2; Profile 3 > Profile 1).

**Figure 2:**
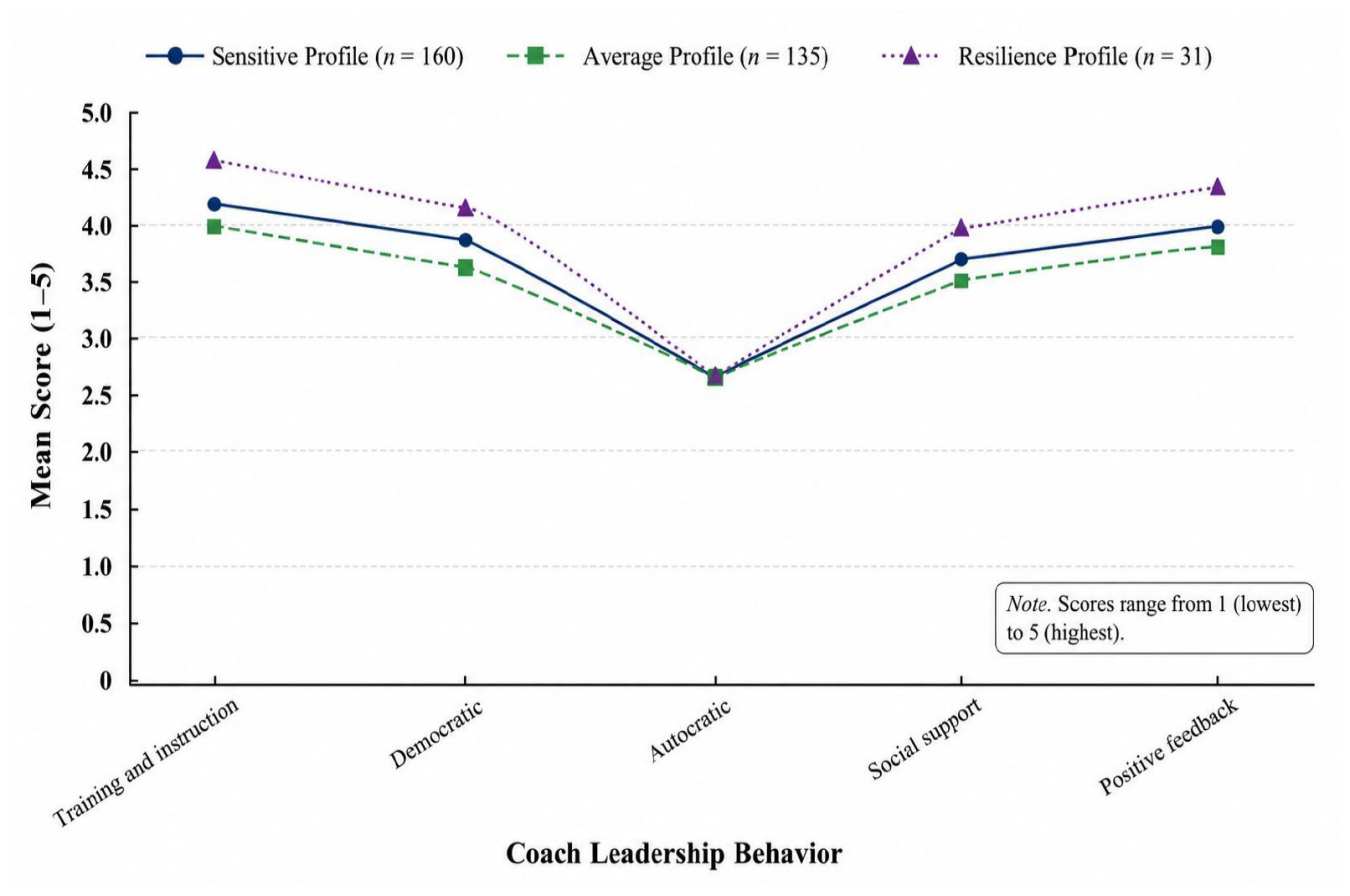
Preference of female basketball players with different personality profiles for coaches' leadership behavior.

As shown in Figure 2, female basketball players with different personality profiles exhibited a nonlinear (“V”-shaped) pattern in their preferences for coaching leadership behaviors, indicating a cross-profile convergence characterized by a “high-medium-low” distribution. Although the three personality profiles differed in their personality characteristics, they showed a highly consistent ranking of preferences for coaching leadership behaviors. Training and instruction behaviors were the most preferred, followed by positive feedback, democratic behaviors, social support behaviors, and autocratic behaviors.

### 3.3 Differential associations between personality traits and leadership behavior preferences across profiles

To examine the associations between the Big Five personality traits and preferences for coaches‘ leadership behaviors across the latent personality profiles, multiple linear regression analyses using the Enter method were conducted separately for each profile. The five personality traits were entered simultaneously as independent variables, and preferences for the five dimensions of coaches' leadership behaviors were treated as dependent variables. The multicollinearity diagnostics are presented in Table 8. Prior to model estimation, multicollinearity diagnostics were performed. Across all three personality profiles, variance inflation factor (VIF) values were below 2.0 and tolerance values exceeded 0.50, indicating that multicollinearity was not a concern and that the assumptions for regression analysis were satisfied.

**Table 8:** Multicollinearity diagnostics for regression models within each profile.

Personality trait	“Sensitive” profile (*n* = 160)		“Average” profile (*n* = 135)		“Resilience” profile (*n* = 31)	
	VIF	Tolerance	VIF	Tolerance	VIF	Tolerance
Extraversion	1.170	0.855	1.023	0.978	1.346	0.743
Agreeableness	1.263	0.792	1.022	0.978	1.596	0.626
Conscientiousness	1.219	0.820	1.017	0.983	1.664	0.601
Neuroticism	1.071	0.934	1.016	0.984	1.717	0.582
Openness	1.178	0.849	1.024	0.977	1.298	0.770

The regression results for the Sensitive profile are presented in Table 9. For the “Sensitive” profile, agreeableness positively predicted preferences for training and instruction behavior (β = 0.39, *p* = 0.01), whereas conscientiousness negatively predicted preferences for training and instruction behavior (β = −0.29, *p* = 0.02). Conscientiousness also negatively predicted preferences for autocratic behavior (β = −0.42, *p* = 0.03) and social support behavior (β = −0.22, *p* = 0.01). In addition, agreeableness (β = 0.34, *p* = 0.04) and neuroticism (β = 0.48, *p* < 0.001) positively predicted preferences for positive feedback behavior.

**Table 9:** Multiple linear regression results for the “sensitive” profile (*n* = 160).

Personality trait	Training and instruction		Democratic		Autocratic		Social support		Positive feedback	
	β	*p*	β	*p*	β	*p*	β	*p*	β	*p*
Extraversion	−0.03	0.83	0.73	0.40	0.15	0.35	0.16	0.06	0.11	0.14
Agreeableness	0.39	0.01	0.15	0.10	−0.27	0.22	0.24	0.01	0.34	0.04
Conscientiousness	−0.29	0.02	−0.13	0.15	−0.42	0.03	−0.22	0.01	−0.13	0.12
Neuroticism	0.13	0.22	−0.50	0.55	0.20	0.90	0.08	0.33	0.48	0.00
Openness	0.15	0.19	0.18	0.07	−0.07	0.67	0.04	0.61	0.09	0.24
*R* ^2^	0.082		0.057		0.072		0.091		0.140	
Adjusted *R*^2^	0.052		0.026		0.042		0.062		0.112	
F	2.747^*^		1.853		2.381^*^		3.089^*^		5.002^**^	

^*^p < 0.05; ^**^p < 0.01.

The regression results for the Average profile are presented in Table 10. For the “Average profile, extraversion positively predicted preferences for democratic behavior (β = 0.32, *p* = 0.04) and positive feedback behavior (β = 0.40, *p* = 0.003), whereas neuroticism positively predicted preferences for autocratic behavior (β = 0.35, *p* = 0.03).

**Table 10:** Multiple linear regression results for the “average” profile (*n* = 135).

Personality trait	Training and instruction		Democratic		Autocratic		Social support		Positive feedback	
	β	*p*	β	*p*	β	*p*	β	*p*	β	*p*
Extraversion	0.19	0.12	0.32	0.04	0.01	0.95	0.08	0.39	0.40	0.00
Agreeableness	0.19	0.27	−0.07	0.74	−0.13	0.15	0.05	0.58	0.01	0.92
Conscientiousness	−0.02	0.91	0.05	0.78	0.02	0.87	−0.04	0.63	−0.07	0.40
Neuroticism	0.12	0.38	0.19	0.26	0.35	0.03	0.15	0.10	−0.01	0.87
Openness	−0.06	0.65	−0.16	0.37	−0.04	0.67	0.01	0.88	−0.02	0.82
*R* ^2^	0.033		0.046		0.054		0.033		0.071	
Adjusted *R*^2^	−0.005		0.009		0.017		−0.005		0.035	
F	0.879		1.238		1.474		0.871		1.962	

The regression results for the Resilience profile are presented in Table 11. For the “Resilience” profile, conscientiousness positively predicted preferences for social support behavior (β = 0.40, *p* = 0.04). However, the sample size for this profile was relatively small (*n* = 31), which may have reduced statistical power and affected the stability of the regression coefficients. Therefore, these findings should be interpreted with caution and regarded as exploratory.

**Table 11:** Multiple linear regression results for the “resilience” profile (*n* = 31).

Personality trait	Training and instruction		Democratic		Autocratic		Social support		Positive feedback	
	β	*p*	β	*p*	β	*p*	β	*p*	β	*p*
Extraversion	0.47	0.13	0.27	0.54	0.87	0.52	0.24	0.19	0.04	0.87
Agreeableness	−0.30	0.50	0.21	0.74	0.01	0.99	−0.18	0.17	0.15	0.54
Conscientiousness	0.21	0.63	−0.04	0.96	−0.09	0.63	0.40	0.04	−0.06	0.80
Neuroticism	−0.12	0.73	−0.45	0.39	0.24	0.18	−0.13	0.52	−0.26	0.32
Openness	−0.11	0.73	−0.39	0.40	−0.23	0.18	−0.04	0.85	−0.23	0.30
*R* ^2^	0.137		0.100		0.270		0.271		0.094	
Adjusted *R*^2^	−0.035		−0.080		0.124		0.125		−0.084	
F	0.795		0.558		1.852		1.855		0.516	

Overall, the explained variance (R^2^) of the regression models was modest, which is consistent with previous research in sport psychology indicating that leadership preferences are influenced by multiple contextual and situational factors beyond personality traits. Accordingly, personality traits alone are not expected to explain a large proportion of the variance in leadership behavior preferences.

Although some regression models were not statistically significant overall (F-test), several individual predictors showed significant associations with specific leadership behavior dimensions, suggesting that personality traits may exert differential effects on particular leadership preferences.

## 4 Discussion

### 4.1 The “Big Five” personality profiles of Chinese female basketball players

Traditional personality research has primarily adopted a variable-centered approach, which describes group characteristics based on the average level of individual personality traits. However, this approach may not adequately capture the diversity of personality configurations within a population. For example, a score below the group mean on a given trait does not necessarily indicate the absence of that trait. Yin et al. (2020) argued that a person-centered approach based on Big Five personality profiles can better capture personality heterogeneity by examining combinations of traits. Accordingly, the present study used latent profile analysis (LPA) to identify distinct personality profiles among female basketball players from the WCBA and CUBAL, providing a more comprehensive understanding of personality configurations in this competitive context.

The three personality profiles identified in this study showed structural similarity to the profiles reported by Alessandri et al. (2013) in a general population sample. However, there is no universally accepted criterion for determining the optimal number of latent profiles, as model selection depends on multiple factors, including sample characteristics, sample size, and the interpretability of the resulting profiles (Herzberg and Roth, 2006). Research applying LPA to examine personality profiles in team sport athletes remains limited. In the four-profile model of rock climbers reported by Rumbold et al. (2021), the “Healthy” profile (12%) shares several characteristics with the “Resilience” profile (23%) identified in the present study. Both represent relatively small but psychologically adaptive subgroups, which have been associated with more positive interpersonal relationships and greater sport satisfaction (Semeijn et al., 2020).

The proportion of athletes classified into the “Sensitive” profile was higher than that reported in some previous studies involving other athletic populations. This discrepancy may reflect differences in sample characteristics, sport type, measurement instruments, or the latent profile solutions identified across studies. Because these factors were not directly examined in the present study, the reasons for this difference remain speculative and require further investigation.

Previous studies have also shown that person-centered approaches such as LPA can identify psychologically meaningful subgroups associated with differences in behavioral and wellbeing outcomes (Shannon et al., 2021). Although the relative advantages of variable-centered and person-centered approaches require further investigation, the present findings suggest that personality profiling can complement traditional variable-centered analyses by identifying subgroups of athletes with similar personality configurations. Such information may facilitate the development of more targeted psychological assessment and intervention strategies (Howard and Hoffman, 2018).

### 4.2 The influence of personality profiles on preferences for coaches' leadership behaviors

#### 4.2.1 “Sensitive” profile

Among athletes in the “Sensitive” profile, who were characterized by relatively high neuroticism and relatively low conscientiousness and agreeableness, conscientiousness was negatively associated with preferences for training and instruction, social support, and autocratic behaviors. In contrast, agreeableness was positively associated with preferences for training and instruction, social support, and positive feedback behaviors.

Training and instruction behaviors primarily involve providing technical and tactical guidance, whereas social support behaviors focus on addressing athletes' individual needs and fostering positive interpersonal relationships. In team sports, these coaching behaviors may be particularly important because athletes train and compete in highly interdependent environments. One possible interpretation is that athletes with higher neuroticism and lower conscientiousness place greater value on structured coaching behaviors under these conditions.

Recent research has shown that coaching leadership behaviors are associated with athletes' psychological experiences through interpersonal processes within the coach–athlete relationship (Liu et al., 2025). Similar interpretations have also been proposed in previous studies on coaching leadership (Riemer and Toon, 2001). However, the present study did not directly examine these underlying mechanisms, and this interpretation should therefore be considered tentative.

##### 4.2.1.1 Conscientiousness

Conscientiousness is generally associated with goal orientation and includes characteristics such as organization, efficiency, and responsibility. Matsumoto et al. (2000) suggested that conscientiousness may be related to training completion, with lower levels often associated with reduced perseverance and weaker self-regulation. Consistent with this view, Carron et al. (1998) proposed that more task-oriented leadership may be beneficial for individuals with lower conscientiousness. Similarly, previous research has linked conscientiousness to self-regulation and goal management (Laborde et al., 2020), suggesting that athletes with lower conscientiousness may benefit from more structured coaching guidance. However, the present study did not directly evaluate coaching effectiveness, and this interpretation should therefore be viewed with caution.

The present findings also showed a negative association between conscientiousness and preferences for autocratic behavior within the “Sensitive” profile. One possible interpretation is that athletes with relatively lower conscientiousness may be more accepting of directive coaching behaviors when clear guidance is needed to support training and competition. Because the present study assessed leadership preferences rather than coaching outcomes, this explanation remains tentative and requires further investigation.

##### 4.2.1.2 Agreeableness

Agreeableness is generally associated with an interpersonal orientation and includes characteristics such as empathy, cooperativeness, trust, and consideration for others. In the present study, agreeableness was positively associated with preferences for training and instruction, social support, and positive feedback among athletes in the “Sensitive” profile. One possible interpretation is that athletes with higher agreeableness place greater value on supportive interpersonal interactions, making coaching guidance and positive feedback more appealing. Previous research has also suggested that the expression of agreeableness may be influenced by situational factors (Carron et al., 1998). In highly interdependent team environments, supportive coaching behaviors may help reduce emotional strain and facilitate positive coach–athlete interactions, particularly for athletes with relatively higher neuroticism. This interpretation highlights the potential interaction between personality traits and situational factors. However, because these mechanisms were not directly examined in the present study, further research is needed to clarify these relationships.

##### 4.2.1.3 Neuroticism

Positive feedback behaviors primarily involve encouragement, recognition, and reinforcement of athletes' efforts and performance. Previous studies have reported gender-related differences in preferences for coaching behaviors, including positive feedback. However, because the present study included only female athletes, direct comparisons across gender were not possible.

Within the “Sensitive” profile, neuroticism was positively associated with preferences for positive feedback. Neuroticism is generally characterized by emotional instability and heightened sensitivity to negative emotional experiences. This finding is broadly consistent with previous research linking neuroticism to interpersonal experiences within the coach–athlete relationship (Yang et al., 2015). Nevertheless, the present findings should not be interpreted as evidence that neuroticism directly determines coaching preferences.

The “Sensitive” profile represented the largest subgroup (49% of the sample) and was characterized by relatively higher levels of neuroticism than the other profiles. One possible interpretation is that athletes with higher neuroticism place greater value on positive feedback because such feedback has been associated with perceived competence and self-efficacy in previous theoretical work (Finley et al., 2017). However, because self-efficacy and emotion regulation were not assessed in the present study, this explanation remains speculative.

The present findings also suggest that the combination of relatively high neuroticism and relatively low agreeableness may be associated with a stronger preference for positive feedback from coaches. One possible explanation is that positive feedback may facilitate more positive coach–athlete interactions and interpersonal relationships within the team. This interpretation is broadly consistent with the multidimensional model of team cohesion (Allen et al., 2013). However, team cohesion was not measured in the present study, and further research is needed to examine these potential mechanisms.

#### 4.2.2 “Average” profile

##### 4.2.2.1 Extraversion

This study found that among individuals with an “average” profile, characterized by relatively balanced personality traits, extraversion was positively associated with preferences for democratic and positive feedback coaching behaviors. Democratic coaching behaviors refer to the extent to which coaches involve athletes in decision-making and consider their opinions. Previous research has suggested that contextual factors, such as the type of sport, may influence coaching leadership behaviors (Riemer and Chelladurai, 1998). For example, team sports often involve managing larger groups and more complex interactions, which may constrain the use of democratic behaviors, whereas individual sports may allow for greater flexibility in decision-making processes.

The present findings suggest that, among individuals with relatively balanced personality traits, extraversion may be an important factor associated with athletes‘ preferences for both democratic behaviors and positive feedback. One possible interpretation is that characteristics associated with extraversion, such as sociability, assertiveness, and high energy levels, may increase athletes' openness to interpersonal interaction and responsiveness to coaches‘ feedback. This interpretation is broadly consistent with social exchange theory, which proposes that supportive coaching behaviors and positive recognition may facilitate beneficial interpersonal exchanges and enhance athletes' psychological experiences (Cropanzano and Mitchell, 2005). However, the present study did not directly examine social motivation or decision-making processes.

In addition, previous meta-analytic research has shown that extraversion is positively associated with participation in group decision-making (Judge et al., 2002). This may help explain why athletes with higher extraversion show stronger preferences for democratic coaching behaviors. Overall, these findings suggest that even when personality traits are relatively balanced, certain core traits such as extraversion may still play a prominent role in shaping leadership preferences. This observation is consistent with trait activation theory, which emphasizes that the expression of personality traits depends on the interaction between individual characteristics and situational demands (Tett and Burnett, 2003). However, these mechanisms were not directly tested in the present study and should therefore be interpreted with caution.

##### 4.2.2.2 Neuroticism

Compared with the Sensitive profile, in which conscientiousness was negatively associated with autocratic behavior, athletes in the Average profile showed a positive association between neuroticism and preferences for autocratic behavior. Although both groups overall showed relatively low preference for autocratic behavior, this difference suggests that preferences for this leadership style may vary depending on personality configurations.

As a core dimension related to emotional stability, neuroticism may be associated with differences in how athletes respond to emotionally demanding situations. Emotion regulation theory (Gross, 2015) provides one possible framework for interpreting this pattern. For example, individuals with lower neuroticism may process negative situations more efficiently, which can reduce the intensity of emotional and behavioral responses (Brock et al., 2022). However, because emotion regulation strategies were not assessed in the present study, this interpretation remains tentative.

One possible explanation is that athletes with relatively higher neuroticism may place greater value on clear instructions and more structured coaching behaviors. This interpretation is consistent with previous research suggesting that neuroticism is associated with increased sensitivity to emotionally demanding contexts. Although (Amaro and Brandão 2023) reported anxiety-related characteristics in female athletes, the present study did not assess anxiety or include male athletes, and therefore no gender-based comparisons can be made.

Overall, the present findings extend previous research by examining neuroticism within a person-centered framework and suggest that athletes with different personality profiles may differ in their preferences for coaching behaviors. These findings may offer preliminary implications for more individualized coaching approaches; however, such applications should be interpreted with caution given the correlational nature of the data.

#### 4.2.3 “Resilience” profile

Within the “Resilience” profile, conscientiousness was positively associated with preferences for social support; however, the overall regression model was not statistically significant, and this finding should therefore be interpreted as exploratory.

Exploratory analyses further indicated that, in contrast to the “Sensitive” profile, conscientiousness was positively associated with preferences for coaches' social support behaviors within the “Resilience” profile. This pattern is consistent with previous research suggesting that conscientiousness is linked to more positive coach–athlete relationships and greater commitment to shared goals (Cook et al., 2021; Jackson et al., 2011). One possible explanation is that athletes with higher conscientiousness may place greater value on supportive coaching behaviors that facilitate communication, coordination, and sustained engagement within the coach–athlete relationship.

Although athletes in the “Resilience” profile also exhibited relatively higher levels of agreeableness and extraversion than those in the “average” profile, these traits were not significantly associated with coaching behavior preferences. This may suggest that the relationships between individual traits and leadership preferences vary across personality configurations, such that the influence of a given trait depends on the broader profile in which it is embedded. This interpretation aligns with the person-centered perspective underlying latent profile analysis, which emphasizes that combinations of traits may provide additional explanatory value beyond individual traits considered in isolation. However, because interactions among traits were not directly tested in the present study, these mechanisms remain speculative.

Overall, these findings highlight the potential value of a person-centered approach in understanding individual differences in athletes' preferences for coaching behaviors. Rather than assuming uniform effects of personality traits across individuals, latent profile analysis suggests that similar trait levels may be associated with different preferences depending on the overall personality configuration. Given the relatively small size of this subgroup (*n* = 31), these findings should be considered preliminary and require replication in larger samples.

### 4.3 Practical implications for coaching practice

For athletes classified within the “Sensitive” profile, the present findings suggest relatively stronger preferences for structured coaching behaviors, including training and instruction, positive feedback, and social support. Accordingly, coaches may consider providing clear task expectations, constructive feedback, and consistent interpersonal support when working with athletes exhibiting similar personality characteristics.

Athletes in the “average” profile demonstrated preferences for both democratic coaching behaviors and positive feedback. Coaches may therefore consider incorporating opportunities for athlete involvement in decision-making while maintaining timely and constructive feedback during training and competition.

For athletes in the “Resilience” profile, conscientiousness was positively associated with preferences for social support behaviors. This suggests that supportive coaching interactions may be particularly well received by athletes with this personality profile. Coaches may therefore consider maintaining supportive communication and collaborative relationships when working with similar athletes.

It is important to note that the practical implications proposed in this study are based on athletes' reported preferences for coaching behaviors rather than direct evaluations of coaching interventions or their effectiveness. Accordingly, these recommendations should be considered preliminary and interpreted with caution. Coaches may take athletes' personality configurations into account when selecting leadership approaches, as this may help align coaching behaviors with athletes' preferences.

Future longitudinal and experimental research is needed to determine whether aligning coaching behaviors with athletes' personality profiles leads to improvements in athlete wellbeing, coach–athlete relationships, and performance outcomes.

## 5 Limitations

It should be acknowledged that this study has several limitations. First, this study employed a cross-sectional design; therefore, no causal inferences can be drawn regarding the relationships between personality traits and preferences for coaches' leadership behaviors. Longitudinal and experimental studies are needed to examine the stability of personality traits and the directionality of these associations over time.

Second, participants were recruited from WCBA and CUBAL teams using convenience sampling. Although these athletes represent a relatively high level of competition in Chinese women's basketball, this sampling strategy may have introduced selection bias and limited the generalizability of the findings.

Third, all variables were assessed using self-administered questionnaires, which may have been influenced by social desirability, recall bias, and individual response tendencies. In addition, because both predictor and outcome variables were collected from the same participants at a single time point, common method variance cannot be fully ruled out, despite the use of standardized scales and anonymous data collection procedures. Future research could integrate athletes' self-reports with coach ratings, peer evaluations, and behavioral observations to enhance measurement validity.

Fourth, the “Resilience” profile included only 31 participants, resulting in limited statistical power for subgroup analyses. Therefore, findings related to this profile should be considered preliminary and require validation in larger samples.

Fifth, although this study focused on the association between personality traits and preferences for coaching leadership styles, several potentially important variables were not included in the analysis, such as competition experience, playing position, training load, coach characteristics, the quality of the coach–athlete relationship, and team atmosphere. These factors may influence athletes' leadership preferences and should be incorporated into future multivariate models.

Sixth, the Sport Leadership Scale assesses athletes' preferences for coaches' leadership behaviors rather than actual coaching practices. Therefore, the findings reflect subjective preferences rather than objectively observed behaviors. Future research should combine athletes' preferences with coaches' self-reports and observational assessments to examine the alignment between preferred and actual leadership behaviors.

Finally, although some regression models did not reach overall statistical significance, certain individual predictors were significantly associated with specific coaching leadership behaviors. These findings should therefore be interpreted with caution and considered exploratory until validated in larger, independent samples.

## Data Availability

The original contributions presented in the study are included in the article/Supplementary material, further inquiries can be directed to the corresponding author.
