# Supplementary material for: Personality profiles and coaching leadership preferences among Chinese female basketball players: a latent profile analysis
Source: Front Psychol. 2026 Sep 10;17:1909620. doi: 10.3389/fpsyg.2026.1909620 (PMC13600989; doi:10.3389/fpsyg.2026.1909620)
Supplement: Supplementary file 1 [file Table_1.DOCX]

# 知情同意书（问卷调查研究）

研究题目：

我国女子篮球运动员对教练员领导行为的偏好：教练员性别的交互作用

研究单位：

XXXX大学（学院名称）

研究负责人：

XXX

联系方式：

电子邮箱：XXXX@XXXX

一、研究目的

本研究旨在了解我国女子篮球运动员对不同教练员领导行为的偏好情况，并探讨教练员性别在其中可能产生的影响。研究结果有助于深化对运动情境中教练—运动员关系的理解，并为教练实践与运动员培养提供参考。

二、研究内容与参与方式

您将被邀请填写一份关于教练员领导行为感知与偏好的问卷。问卷为匿名形式，预计填写时间约为10–15分钟，仅需完成一次。

三、自愿参与与退出权利

您的参与完全出于自愿。您可以选择不参加本研究，或在填写问卷过程中随时退出，而无需说明原因，且不会因此受到任何不利影响。本研究的参与与您的训练、比赛、队内评价或选拔等事项无任何关联。

四、潜在风险与不适

本研究不涉及身体风险。部分问题可能会引发您对训练或比赛经历的回顾，若您在回答过程中感到不适，可选择跳过相关问题或立即退出问卷。

五、研究受益

您参与本研究不会获得直接的个人收益，但研究结果可能有助于改进教练领导方式，促进运动员身心发展，并为相关学术研究和实践提供依据。

六、隐私保护与数据保密

本研究采用匿名问卷调查方式，不收集您的姓名、联系方式或任何可识别个人身份的信息。问卷通过在与主教练事先沟通后进行分发，但教练员无法获知谁参与了研究，也无法查看任何个人或整体问卷数据。所有数据仅用于学术研究，由研究团队妥善保管，严格遵守数据保密与隐私保护原则，不会向无关第三方披露。

七、伦理审批

本研究已提交至XXXX大学伦理委员会进行伦理审查，并在获得批准后开展（伦理批准编号：XXXX，批准日期：XXXX）。

八、知情同意声明

我已阅读并理解上述研究说明，知晓本研究的目的、内容、可能风险及我的相关权利。我自愿参加本研究，并同意研究者在保护我个人隐私的前提下使用我的问卷数据用于学术研究。

□ 我同意参与本研究

（电子问卷中，勾选/点击“同意”即视为知情同意）
